# Parliament and the Professions

**Published:** 1921-04-16

**Authors:** 


### Parliament and the Professions.

### Yokohama Naval Hospital.

The Secretary to the Admiralty, replying to a question as to the necessity for the Royal Naval Hospital at Yokohama, said that it is required for the reception of convalescent cases from Hong Kong to remove them from the period of damp and hot weather there. Its abolition would result in a larger number of cases being invalided home from the China Station, with the consequent inconvenience and expense. The questioner, Commander Bellairs, pointed out that the maximum number using the eighty or ninety beds in recent years was eight, and that in 1920 not a single patient was accommodated. Mr. Avery said he would inquire further, and see if any economy can be effected.

### Ex-Soldiers and Tropical Diseases.

The Minister of Pensions stated that the question of the treatment of men disabled in former wars, suffering from certain tropical diseases, in Government clinics is now under consideration.

